# The WRB Subunit of the Get3 Receptor is Required for the Correct Integration of its Partner CAML into the ER

**DOI:** 10.1038/s41598-019-48363-2

**Published:** 2019-08-15

**Authors:** Hugo J. F. Carvalho, Andrea Del Bondio, Francesca Maltecca, Sara F. Colombo, Nica Borgese

**Affiliations:** 1 Consiglio Nazionale delle Ricerche Institute of Neuroscience and BIOMETRA Department, Università degli Studi di Milano, I-20129 Milan, Italy; 2 Division of Neuroscience, Ospedale San Raffaele, I-20132 Milan, Italy; 3 Present Address: Institute of Bioengineering, School of Life Sciences, Ecole Polytechnique Fédérale de Lausanne (EPFL), 1015 Lausanne, Switzerland

**Keywords:** Organelles, Membrane trafficking

## Abstract

Calcium-modulating cyclophilin ligand (CAML), together with Tryptophan rich basic protein (WRB, Get1 in yeast), constitutes the mammalian receptor for the Transmembrane Recognition Complex subunit of 40 kDa (TRC40, Get3 in yeast), a cytosolic ATPase with a central role in the post-translational targeting pathway of tail-anchored (TA) proteins to the endoplasmic reticulum (ER) membrane. CAML has also been implicated in other cell-specific processes, notably in immune cell survival, and has been found in molar excess over WRB in different cell types. Notwithstanding the stoichiometric imbalance, WRB and CAML depend strictly on each other for expression. Here, we investigated the mechanism by which WRB impacts CAML levels. We demonstrate that CAML, generated in the presence of sufficient WRB levels, is inserted into the ER membrane with three transmembrane segments (TMs) in its C-terminal region. By contrast, without sufficient levels of WRB, CAML fails to adopt this topology, and is instead incompletely integrated to generate two aberrant topoforms; these congregate in ER-associated clusters and are degraded by the proteasome. Our results suggest that WRB, a member of the recently proposed Oxa1 superfamily, acts catalytically to assist the topogenesis of CAML and may have wider functions in membrane biogenesis than previously appreciated.

## Introduction

**Figure 1:**
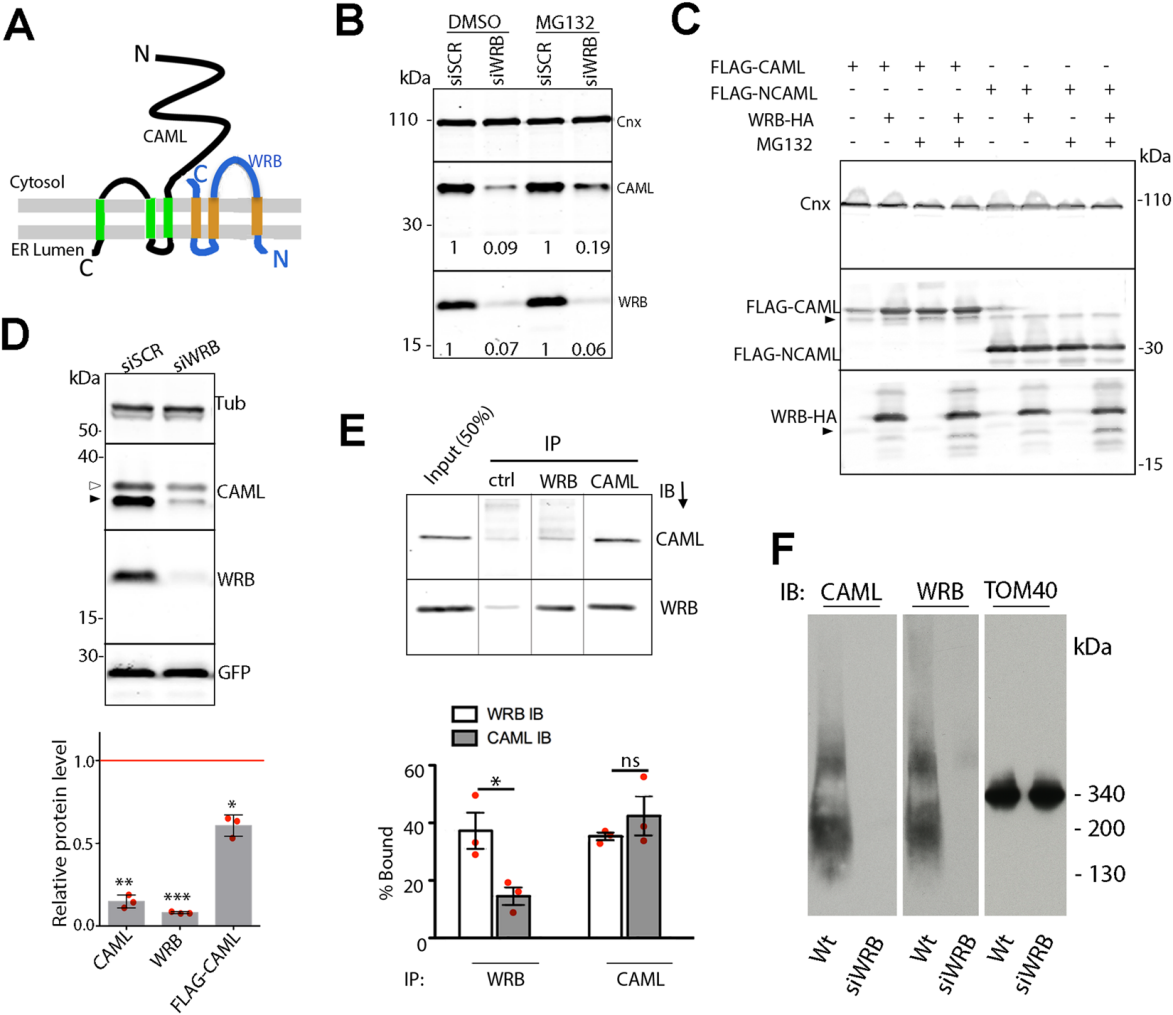
Relationship of CAML to WRB in silenced, overexpressing and wild-type cells. (**A**) Cartoon of the WRB/CAML complex. (**B**) WRB depletion causes proteasomal degradation of CAML. Cells, exposed to scrambled (siSCR) or WRB targeted siRNA (siWRB), and treated with MG132 or vector for 6 h, were analysed by SDS-PAGE/IB with the indicated antibodies. The fold-variation of CAML levels in WRB silenced cells *vs* control is shown below the lanes. (**C**) Destabilization of CAML by insufficient WRB requires its membrane-binding domain. Cells were transfected with FLAG-CAML or FLAG-NCAML together with WRB-HA where indicated, and then analysed by SDS-PAGE/IB. Black arrowheads indicate endogenous WRB or CAML. (**D**) The levels of transfected FLAG-CAML are reduced in WRB-silenced cells. Silenced or control cells, co-transfected with FLAG-CAML and EGFP, were analysed by SDS-PAGE/IB with the indicated antibodies. White and black arrowheads indicate FLAG-CAML and endogenous CAML, respectively. The fold-change of FLAG-CAML levels in WRB-silenced *vs* siSCR-treated cells is shown in the graph as mean ± s.d. of three independent experiments, each analysed in duplicate. GFP and Tubulin were used for the normalization of FLAG-CAML and endogenous CAML, respectively. Significance of differences between silenced and control cells was determined with the paired two-tailed t-test after logarithmic transformation of the data (*, **, ***p = 0.015, 0.0062, 0.0003, respectively). (**E**) Analysis of association of endogenous WRB and CAML by Co-Ip. Cleared lysate was exposed to anti-WRB, anti-CAML or irrelevant IgG (ctrl) under non-denaturing conditions. Immunoprecipitated proteins were analysed by SDS-PAGE/IB with the indicated antibodies. The % immunoprecipitated proteins is shown in the graph (mean of 3 independent experiments ± s.e.m.). Significance of differences between WRB and CAML Ip efficiency with each antibody was evaluated by unpaired two-tailed t test (*p = 0.031; ns, non-significant). Vertical lines separate lanes from the same blot acquired together. (**F**) BN-PAGE/IB analysis of endogenous WRB/CAML. TOM40 was probed both as loading control and as size marker. The positions of mitochondrial complex II and IV (130 and 200 kDa) are also indicated. Original, uncropped blots of panels (B–F) are shown in Supplementary Fig. S4A–E, respectively.

Tryptophan Rich Basic Protein (WRB) and Calcium Modulating Cyclophilin Ligand (CAML), the mammalian functional homologues of yeast Get 1 and 2, respectively, constitute the receptor for the Get3 homologue Transmembrane Recognition Complex subunit of 40 kDa (TRC40)^1–3^. This cytosolic ATPase captures tail-anchored (TA) proteins in the cytosol and delivers them to the Endoplasmic Reticulum (ER) membrane by interacting with the WRB/CAML receptor (see^4,5^ for recent reviews). Each subunit is predicted to have three Transmembrane segments (TM); this topology has been experimentally confirmed for Get1^6^, while the disposition of CAML’s TMs has not yet been directly investigated. However, it is established that the N-terminal domain of CAML is cytosolic, and involved in the capture of TRC40, while the predicted three TMs are close to the C-terminus. This C-terminal region is required for the interaction with WRB (Fig. 1A)^2,3^, indicating that complex formation involves interactions between the subunits’ TMs.

As is the case for many multisubunit complexes, the levels of each subunit of the WRB/CAML complex are strictly dependent on the presence of the other one^7,8^. This mutual dependence fits with the idea that the two are associated in a complex of defined stoichiometry, and that each becomes unstable in the absence of its partner. Indeed, the unshielded TMs of membrane-embedded unassembled polypeptides may serve as cues for the engagement of surveillance pathways that preserve proteostasis by eliminating potentially toxic orphaned subunits (reviewed in^9^). In the case of CAML, however, our previous results indicated that the ratio of CAML to WRB varies in different cell types, and that, in all the cases analysed, CAML is in stoichiometric excess^7^. The purpose of this excess CAML might, on the one hand, be linked to TA protein biogenesis, e.g., the efficiency of recruitment of TRC40-TA complexes to the ER could be increased by additional CAML subunits, which are responsible for the initial capture event^3^; on the other hand, excess CAML could be involved in processes independent from TA protein insertion, notably in the immune system (reviewed in^10^), as suggested by a recent study^11^. Whatever its role, it is difficult to reconcile this stoichiometric imbalance with a mechanism of stabilization of CAML based on the shielding of its TMs by WRB on a 1:1 basis.

Here, we have investigated how WRB affects CAML biogenesis and stability. We provide additional evidence supporting the molar excess of CAML over WRB, and experimentally verify the three-TM topological prediction of CAML’s C-terminal domain; this topology, however, is attained only in the presence of WRB. When WRB levels are insufficient, CAML’s TMs are unable to insert into the bilayer correctly; instead, two incorrectly integrated topoforms are generated, which aggregate and undergo proteasome-mediated degradation. We suggest that WRB may function catalytically as a chaperone/insertase to allow the correct topogenesis of its partner, and that it may play a wider role in membrane biogenesis than assisting insertion of TA proteins *via* the TRC40 pathway.

## Results

### Insufficient levels of WRB drive TM-dependent proteasomal degradation of CAML

CAML is known to interact with WRB *via* its C-terminal region, which contains three predicted TMs; the cytosolic N-terminal portion is involved in the capture of TRC40-TA substrate complexes^3^, (Fig. 1A). Silencing WRB in cultured cells or genetic ablation of the *WRB* gene in mice causes a parallel depletion of CAML^7,12^. As reported in Fig. 1B and Supplementary Fig. S4, the decrease of endogenous CAML caused by knock-down of WRB was partially reversed by the proteasomal inhibitor MG132. Conversely, the levels of transfected full-length FLAG-tagged CAML were increased by co-transfection with WRB-HA or by treatment with MG132 (Fig. 1C and Supplementary Fig. S4). Thus, when WRB levels are insufficient, CAML is prone to proteasomal degradation. In contrast, the levels of a truncated form of CAML lacking the C-terminal membrane binding region, (called FLAG-NCAML) were affected neither by the co-transfection of WRB-HA nor by MG132 (Fig. 1C and Supplementary Fig. S4), indicating that destabilization of full-length CAML depends on its membrane-interacting region.

We next asked how WRB depletion would affect expression of FLAG-CAML transfected alone. As shown in Fig. 1D and Supplementary Fig. S4, silencing of WRB significantly reduced the levels of the transfected protein over its already low levels in control cells not expressing exogenous WRB (Fig. 1C,D), suggesting that endogenous WRB can increase the stability of excess CAML. Based on the comparison with known amounts of recombinant WRB and CAML, our previous work, indeed, indicated that in cultured cells and rat liver microsomes, CAML is in 4–7 molar excess over WRB^7^. To further investigate the relationship between the two proteins, we performed co-immunoprecipitation (Co-Ip) experiments with anti-WRB and anti-CAML antibodies under native conditions (Fig. 1E and Supplementary Fig. S4)). While the anti-CAML antibody pulled down equivalent percentages of CAML and WRB, the anti-WRB antibody was more efficient in recovering WRB than CAML. These results are in agreement with our previous conclusion that in cells WRB is quantitatively associated with CAML^7^, and suggest, instead, that a portion of CAML is not bound, or only loosely bound, to WRB.

We then applied Blue Native (BN)- polyacrylamide gel electrophoresis (PAGE) to investigate whether excess CAML is in complexes separate from those containing WRB. Contrary to our expectation, WRB and CAML migrated together as two heterogeneous populations, in which the relative intensities of the two components appeared to remain constant: a major, very broad band, centred at ~200 kDa, presumably containing at least two different complexes, and a less abundant one, centred at ~500 kDa (Fig. 1F and supplementary Fig. S4). These observations are consistent with the self-association of a basic core consisting of CAML and WRB, and/or the association of this core with other proteins, which remain to be identified. Our attempts to detect TRC40, an expected component of at least some of the complexes, failed, likely due to the poor performance of our antibody on the native protein on blots. The results of the BN-PAGE analysis, combined with those of panel E, suggest that WRB and CAML exist in supramolecular complexes, within which CAML may engage in interactions of different strength.

### When WRB levels are insufficient, CAML forms ER-associated clusters that increase in size upon proteasome inhibition

**Figure 2:**
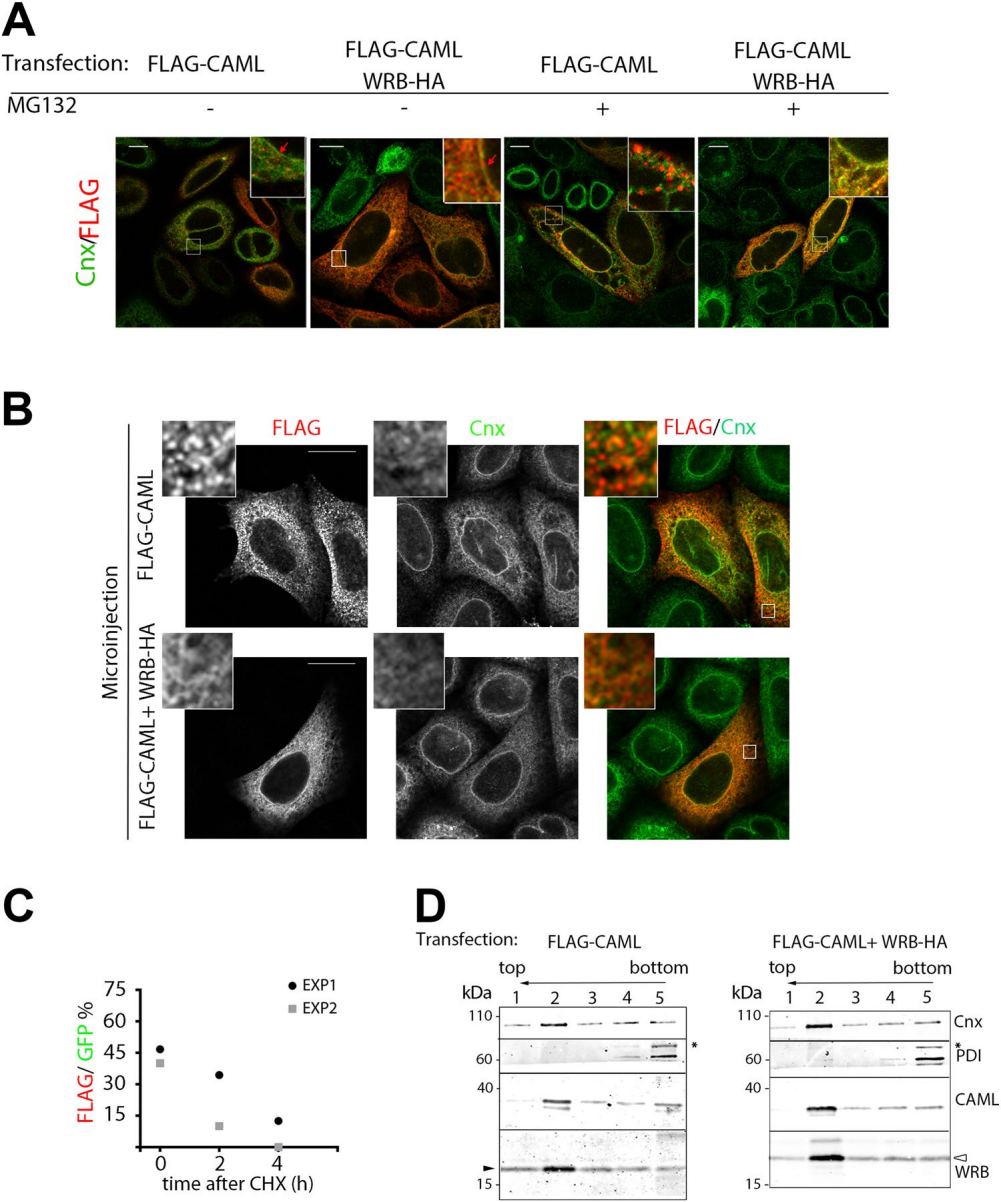
In the absence of WRB, CAML forms ER membrane-associated foci, which are rapidly eliminated. (**A**) HeLa cells were co-transfected with FLAG-CAML and either WRB-HA or an empty plasmid and then treated with MG132 or vector for 6 h. After fixation and staining with anti-FLAG and anti-Cnx antibodies, cells were imaged by confocal microscopy. Merged images of the two channels are shown; the corresponding single channel images are shown in Fig. S1A. Scale bars 10 μm; insets, x4. (**B**) Acutely expressed FLAG-CAML forms foci when expressed alone. Cells were microinjected with the FLAG-CAML plasmid together with either the WRB-HA plasmid or an empty vector, as indicated, and incubated for 2 h before being processed for immunofluorescence with anti-FLAG and anti-Cnx antibodies. Scale bars 10 μm; insets, x6. **(C**) FLAG-CAML foci are rapidly cleared from microinjected cells. Cells were microinjected with the FLAG-CAML plasmid together with pEGFPN1 and incubated for 2.5 h. Cells were then either fixed immediately or treated with CHX for 2 h or 4 h before being processed for immunofluorescence with anti-FLAG antibodies. The time course of decrease in the percentage of GFP-positive cells positive also for FLAG is plotted individually for two independent experiments (number of GFP-positive cells in experiment 1 and 2, respectively: time 0, 75 and 25; 2 h, 32 and 30; 4 h, 32 and 18). Representative images for these experiments are shown in Fig. S1B. **(D)** FLAG-CAML is integrated into membranes also when expressed in the absence of WRB-HA. Membrane fractions of HeLa cells co-transfected with FLAG-CAML and either WRB-HA or an empty plasmid were treated with Na_2_CO_3_ and subjected to floatation through alkaline sucrose gradients. The collected fractions were analyzed by immunoblotting with the antibodies indicated to the right of the blots. WRB, CAML, and Cnx accumulated at the 1.6 M–0.25 M sucrose interface (fraction 2), while the soluble protein PDI remained in the load zone. The black (left) and open (right) arrowheads indicate the endogenous and transfected HA-tagged WRB, respectively. The asterisk on the right of the PDI panels indicates a non-specific band. Original, uncropped blots are shown in Supplementary Fig. S5.

Since unstable proteins are often prone to aggregation, we applied confocal microscopy to analyse the intracellular distribution of FLAG-CAML, expressed alone or together with WRB-HA (Fig. 2A, and Supplementary Fig. S1A). FLAG-CAML, expressed alone, had an intracellular distribution grossly similar to that of calnexin (Cnx), used as ER marker; however, closer inspection revealed that the transfected protein tended to concentrate in foci, which were distributed along the ER network and nuclear envelope, and within which Cnx did not accumulate. MG132 treatment caused a dramatic increase in the size and brightness of these clusters. In contrast, when FLAG-CAML was co-transfected with WRB-HA, it showed a homogeneous reticular and nuclear envelope staining, superimposable on Cnx, a pattern not appreciably affected by MG132 (Fig. 2A). FLAG-CAML foci were observed also in cells that were microinjected with the corresponding plasmid (Fig. 2B and Supplementary Fig. S2B). The foci were seen as soon as the cDNA product became detectable, at 2 h after the microinjection, but not when the FLAG-CAML plasmid was co-microinjected with one specifying WRB-HA. Thus, when expressed alone, CAML clusters rapidly after its synthesis. To investigate whether the FLAG-CAML clusters are susceptible to degradation, cells, microinjected with the FLAG-CAML plasmid together with one coding for EGFP, were allowed to express the encoded proteins for 2 h and then treated with cycloheximide (CHX); the percentage of EGFP positive cells expressing also FLAG-CAML was scored at different times after addition of the inhibitor. As shown in Fig. 2C and Supplementary Fig. S1B, FLAG-CAML was rapidly cleared from the microinjected cells, indicating that the clusters are susceptible to degradation. Furthermore, cluster formation was inherent to “solitary” CAML and not to WRB-HA. Although the levels of WRB-HA increased when co-transfected with FLAG-CAML or upon MG132 treatment (Supplementary Figs S2A and S7), “solitary” WRB-HA, expressed from the corresponding microinjected plasmid, didn’t form detectable clusters (Supplementary Fig. S2B).

We asked whether the FLAG-CAML foci generated upon its “solitary” transfection contained uninserted or dislocated molecules rather than clusters of ER-inserted polypeptide. To investigate the relationship of FLAG-CAML to the lipid bilayer, we treated a membrane fraction isolated from the transfected cells with 0.1 M Na_2_CO_3_, and then allowed the treated membranes to float through an alkaline sucrose gradient (Fig. 2D and Supplementary Fig. S5). Similarly to the integral membrane protein Cnx, and to WRB, most of the FLAG-CAML, whether transfected alone or together with WRB-HA, floated out of the load zone and accumulated at the interface between the 1.6 and 0.25 M sucrose layers. Thus, by the most stringent biochemical criterion used to experimentally define integral membrane proteins, FLAG-CAML clusters appear to contain polypeptides that are integral to the lipid bilayer.

### WRB is required for CAML topogenesis

**Figure 3:**
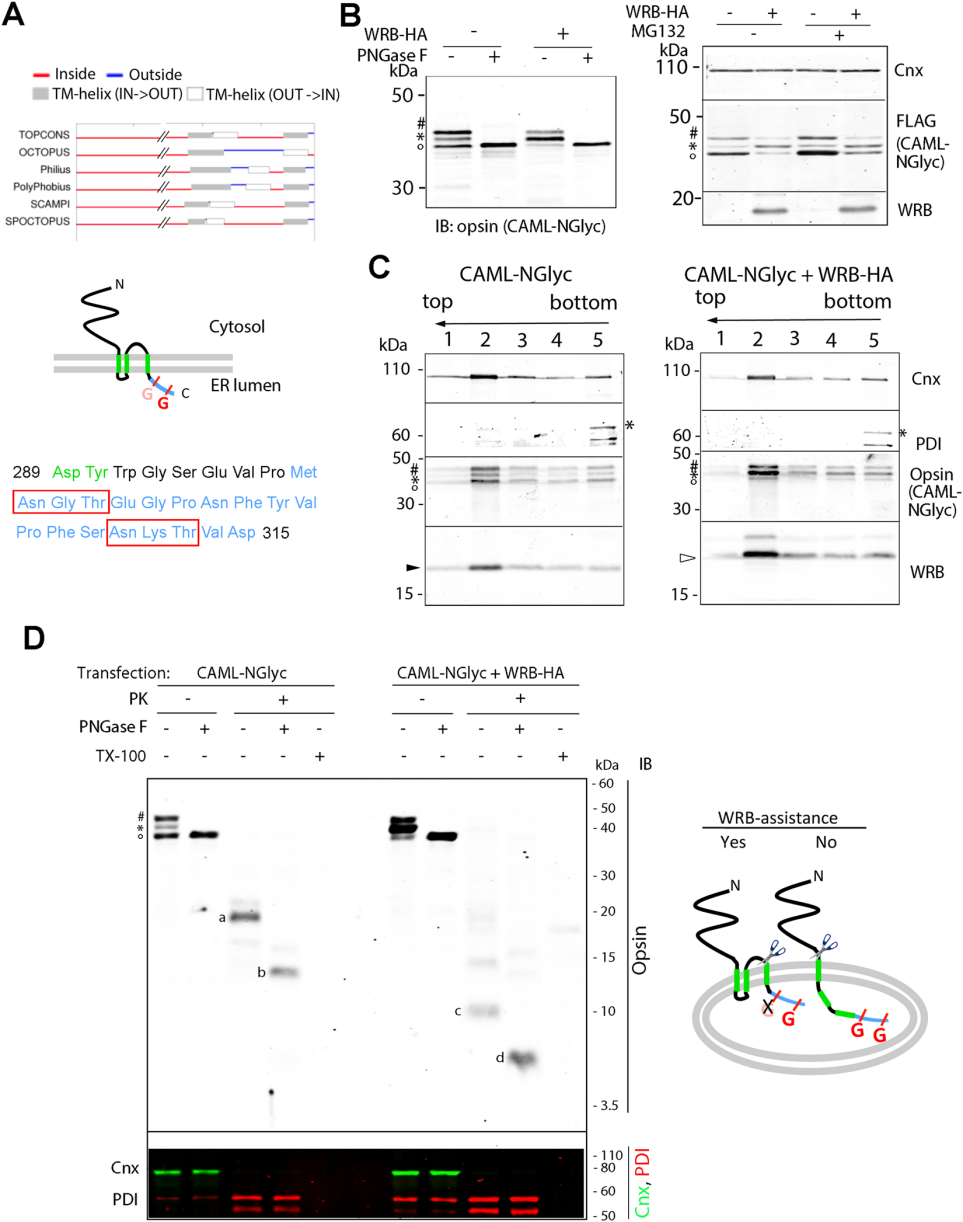
A tagged CAML construct with two N-glycosylation acceptor sites near the C-terminus reports on the topology of CAML expressed in the presence or absence of WRB-HA. (**A**) Predicted CAML topology. Top: Comparison of CAML TMs predicted with different scales (TOPCONS^54^). Middle: The three-TM model for CAML topology predicts that the C-terminus is in the ER lumen. The attached opsin epitope is shown in blue, and its two N-glycosylation sites are indicated (G). The site in pink, because of its proximity to the membrane is expected to remain unused. Bottom: Sequence (blue) of opsin epitope attached to the C-terminal residue of CAML. The last two residues of the predicted third TM are in green. The two N-glycosylation sites are boxed in red. (**B**) Differences in glycosylation of CAML-NGlyc expressed in the presence or absence of WRB-HA. Left: Lysates from cells expressing CAML-NGlyc either alone or together with WRB-HA were treated or not with PNGase F and analysed by SDS-PAGE/IB. Right: Cells were treated with MG132 or vector for 6 h before lysis. o, *, and ^#^ indicate non-glycosylated (0-G), mono-glycosylated (1-G) and di-glycosylated (2-G) forms of CAML-NGlyc, respectively. (**C**) All three CAML-NGlyc forms are associated with membranes. Membrane fractions were treated with Na_2_CO_3_ and subjected to floatation, as in Fig. 2D. Filled and open arrowheads indicate endogenous WRB and WRB-HA, respectively. (**D**) Cells were semi-permeabilized and treated or not with PK followed by PNGase F. One fifth of the unproteolyzed samples was loaded compared to PK-treated ones. The lower panel demonstrates that lumenal PDI is inaccessible to protease, while the cytosolic Cnx epitope is lost after PK treatment. The cartoon depicts the genesis of the a–d bands detected in the blot of panel A. The 8.5 kDa and 5.5 kDa bands c and d are compatible with the three-TM model, while the 20.5 and 12.5 kDa bands a and b have the size expected if both the second and the third TMs are translocated. Original, uncropped blots of panels B left, B right, C and D are shown in Supplementary Fig. S6A–D, respectively.

The rapidity of the formation of FLAG-CAML clusters prompted us to consider the possibility that, when expressed alone, it might encounter problems during the initial stage of its biogenesis involving acquisition of the correct topology of its TMs. Most algorithms predict that CAML has three TMs in its C-terminal region, however, while there is a general consensus on the position of the first and third TM, different predictions are made on the position, and even the existence, of the second one (Fig. 3A, top). The 3-TM model predicts that CAML’s C-terminus is in the ER lumen (Figs 1A and 3A, middle). To experimentally confirm this prediction, we attached an opsin epitope to FLAG-CAML’s C-terminus (Fig. 3A, middle and bottom). The opsin epitope contains two N-glycosylation consensus sites that are potential targets of the lumenal Oligosaccharyltransferase (OST) complex (boxed in Fig. 3A, bottom), hence we refer to the construct as CAML-NGlyc. If the 3-TM model is correct, the second of these sites should be used, while the first one, due to its proximity to the ER membrane, is predicted to be inaccessible to the OST complex^13^. Indeed in many previous studies, in which the opsin sequence was attached immediately downstream of a TM, only a mono-glycosylated protein was generated (e.g.)^14^; thus the opsin sequence can serve both as reporter for translocation to the lumen and as an indicator of the distance of the sequence from the membrane.

Sodium Dodecyl Sulfate (SDS)-PAGE/immunoblot (IB) analysis of lysates from cells transfected with CAML-NGlyc revealed three bands; the two more slowly migrating ones collapsed to the lower molecular weight polypeptide after treatment with Peptide:N-glycosidase F (PNGase F), identifying the three bands (from top to bottom) as di-glycosylated (2-G) mono-glycosylated (1-G), and non-glycosylated (0-G) forms of CAML-NGlyc (Fig. 3B, left panel and Supplementary Fig. S6). Both the 0-G and 2-G bands were stabilized by MG132 (Fig. 3B, right panel), and all three forms were tightly integrated in the ER membrane, as demonstrated by alkaline sucrose gradient floatation analysis (Fig. 3C and Supplementary Fig. S6). When CAML-NGLyc was co-expressed with WRB-HA, the predominant band was the 1G species (left panel, third lane); instead, when CAML-NGLyc was expressed alone, the 0-G and 2-G forms were predominant.

The 1-G form, generated in the presence of transfected WRB-HA, is consistent with the 3-TM model, and rules out the 2-TM topology predicted by some algorithms. This conclusion was further supported by a protease protection assay on semi-permeabilized (SP) cells. The plasma membrane of the transfected cells was selectively permeabilized with digitonin under conditions that leave the ER membrane intact, and the resultant SP cells were exposed to protease K (PK). The sizes of the fragments protected from digestion by the sealed ER membrane were then compared by SDS-PAGE/IB. The inaccessibility of the ER lumen and the accessibility of the cytosolic surface to PK were confirmed by probing for the lumenal protein PDI, and the cytosolic epitope of Cnx, respectively (Fig. 3D, bottom panel, Supplementary Fig. S6). When CAML-NGlyc was co-expressed with WRB-HA, the principal protected fragment (PF) migrated at ~10 kDa (band c in Fig. 3D), and at 5.5 kDa after treatment with PNGase F (band d); this size corresponds closely to the 5.39 kDa fragment (spanning residues 270–315) predicted to be generated from the protein with 3-TM topology (cartoon of Fig. 3D, left structure).

The use of both glycosylation sites in the CAML 2G form suggested that the proximal site could be sufficiently removed from the lumenal face of the ER membrane to become accessible to the OST complex. This supposition was confirmed by protease protection (Fig. 3D and Supplementary Fig. S6). When CAML-NGlyc was transfected alone, the predominant PF ran at 20.5 kDa (Fig. 3D, band a), and was converted to a 12.5 kDa species (band b) after digestion with PNGase F, a size close to the 12.43 kDa PF (residues 210–315) predicted to be generated if the entire region spanning the second and third TM plus the opsin epitope is translocated (cartoon of Fig. 3D, structure on the right). Very low intensity a and b bands were detected in PK-digested samples of CAML-NGlyc co-expressed with WRB-HA, in agreement with the 2G form being a minority component under these conditions.

**Figure 4:**
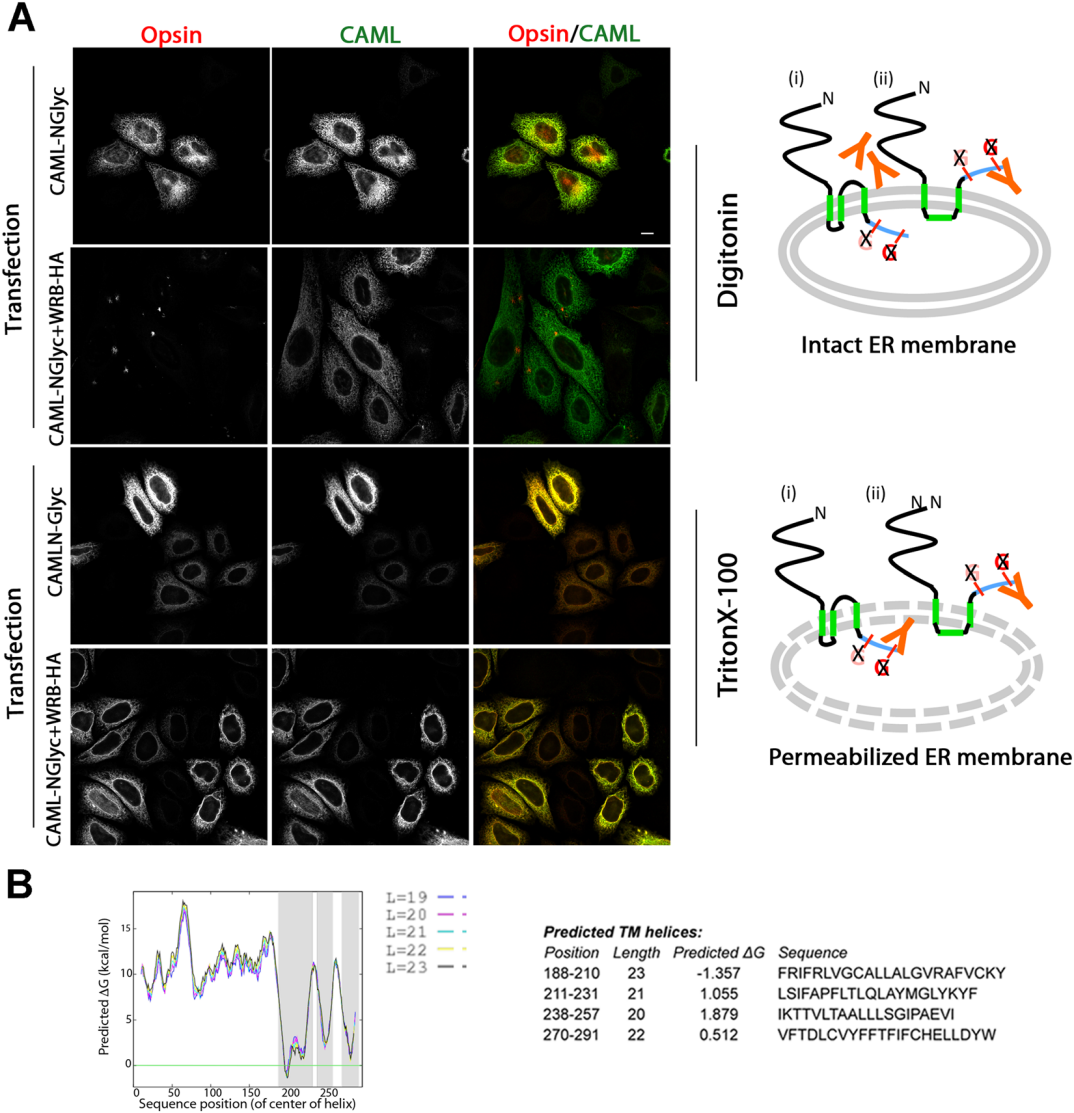
In the absence of WRB-HA, the C-terminus of a portion of CAML-NGlyc molecules is exposed to the cytosol. (**A**) Cells, transfected with CAML-NGlyc either in the presence or absence of WRB-HA, were semi-permeabilized with digitonin before fixation. One set of samples was then processed for immunofluorescence without the addition of other detergents (upper two rows), while the second set of was processed in the presence of TX-100 (lower two rows). Samples were stained with anti-opsin and anti-CAML to visualize the C-terminal opsin epitope and the N-terminal domain of CAML-NGlyc, respectively. Scale bars 10μm. The models on the right illustrate two possible topologies of the 0-G form, distinguishable by the accessibility of the opsin epitope to antibodies: in structure (i), the 0-G form is correctly inserted with the 3-TM topology, but fails to access the OST complex; in structure (ii), TM3 fails translocation, and the opsin epitope is therefore accessible to antibodies (orange) restricted to the cytosol in SP cells. If structure (i) were correct, the opsin epitope would become accessible only in the presence of TX-100 (lower cartoon), as is indeed the case when CAML-NGlyc and WRB-HA are co-expressed. (**B**) Plot of ΔG for membrane integration of CAML sequence. The different colour-coded lines were obtained with different lengths (L) of the sliding window used by the algorithm, as indicated (http://dgpred.cbr.su.se/)^15^. Positive ΔG values predict an unfavourable free energy of membrane integration. Note the unfavourable ΔG of the second predicted TM (either res 211–231 or res 238–257, see text for further details).

The 0-G form could consist in a correctly inserted, 3-TM, protein, which, in the absence of sufficient WRB, is unable to access the OST complex (structure (i) in the cartoons of Fig. 4A); alternatively the C-terminus might fail translocation when WRB is insufficient, leaving the opsin epitope in the cytosol (e.g., structure (ii) in Fig. 4A). In the former case, PK digestion of the samples of CAML-NGlyc transfected alone should have generated a non-glycosylated ~5.5 kDa species (with the mobility of band d), unaffected by PNGase F treatment and of comparable intensity to band a; this species, however, was never detected (Fig. 3D). To confirm the lack of translocation of the C-terminus (structure ii), we probed the accessibility of the opsin epitope by immunofluorescence in SP cells. When CAML-NGlyc was co-expressed with WRB-HA, the opsin epitope was not detected in SP cells, although co-staining with anti-CAML antibodies (which recognize the N-terminal region) demonstrated that the protein was expressed (Fig. 4A, 2nd row). Indeed, after permeabilization of the ER membrane with Triton X-100 (TX100), the opsin epitope became accessible (Fig. 4A, fourth row). In contrast, when CAML-NGlyc was expressed alone, the opsin epitope was revealed regardless of whether the ER membrane was permeabilized with Triton (Fig. 4A, 1st and third rows). The inaccessibility of the ER lumen was confirmed also by the absence of staining of the lumenal protein calreticulin in the SP cells (Fig. S3). Thus, when WRB levels are insufficient, a portion of CAML-NGlyc molecules fails to translocate the C-terminus.

## Discussion

CAML and WRB are the two components of the mammalian TRC40 receptor (corresponding to the Get1/2 complex in yeast), which plays a key role in the insertion of TA proteins into the ER membrane. CAML’s expression strictly depends on the presence of WRB^7,12^. In the present study, we have investigated the mechanism by which WRB affects CAML levels, and show that CAML requires WRB in order to acquire a stable, correctly integrated, conformation in the ER membrane. By glycosylation, antibody and protease accessibility assays, we demonstrate that CAML, when co-expressed with WRB, resides in the membrane with the 3-TM topology and translocated C-terminus postulated by most prediction methods. In contrast, when expressed without additional exogenous WRB, CAML forms clusters that are rapidly cleared, and, importantly, fails to achieve the 3-TM topology. Instead, it is mainly inserted with two alternative topologies: one, in which the entire region comprising the second and third TM is translocated into the ER lumen (referred to here as TMs-translocated), the other, in which the C-terminus, immediately downstream of the third TM, is exposed to the cytosol (referred to here as C-cytosol); the latter form is consistent with a 2-TM topology resulting from the failure of one of the three predicted TMs to integrate into the bilayer.

Unlike the TMs of single-spanning membrane proteins, a sizeable portion of those of polytopic proteins are not sufficiently hydrophobic to be recognized as TMs by the Sec61 translocon^15,16^. Although the moderate hydrophobicity of these TMs is often crucial for their function, it poses difficulties for their one-by-one co-translational insertion into the lipid bilayer *via* the Sec61 translocon. Consequently, the stable integration of these polytopic proteins usually involves the interaction, already during the process of membrane integration, of the weak TMs with other ones of the same polypeptide or of an oligomer partner^17–20^; such inter-helix interactions shield the polar residues of the weak TMs from unfavourable interactions with the lipid bilayer core (reviewed in^16^).

In the case of CAML, while prediction methods unanimously identify the region between residues 188–193 as the start of the first TM, and residues 270–288/291 as the third one, there is a lack of consensus on the second TM, which may be identified as immediately following a short first TM (residues 210/213–230/231), or further downstream (residues 238/240–257/258), or may not be recognized at all (see Fig. 3A, top panel). Both the predicted upstream and downstream second TMs have unfavourable free energy for insertion into the bilayer (Fig. 4B). The third TM also poses a problem for integration: in addition to being poorly hydrophobic, it is so close to the C-terminus that co-translational interaction with the translocon is precluded, and translocation of the C-terminus across the bilayer must necessarily occur post-translationally. The mechanism underlying this process has not, to our knowledge, been investigated either in CAML or in any other polytopic membrane protein with similar topology. The first predicted TM has, instead, a favourable free energy for insertion and is well positioned to interact with SRP and engage the translocon^15^. Thus, CAML insertion presumably begins with the engagement of Sec61 by the first TM, which can exit the translocon’s lateral gate^21^ and serve as a signal anchor. In a stepwise insertion process, the second TM should function as a stop transfer sequence^22^, followed by the post-translational integration of the third TM with concomitant translocation of the C-terminus. In the absence of sufficient WRB, however, this process is not brought to successful completion.

On the basis of our results, we propose that WRB is required for the post-translational insertion of TM3 and translocation of the C-terminus into the ER lumen; failure of this process would underlie the generation of both the C-cytosol and TMs-translocated forms. Analogously to insertion of coupled helices in other polytopic proteins, correct generation of a stable protein would depend on the interaction between the last two TMs of CAML. In the absence of insertion of TM3, TM2 would not be recognized as a transmembrane helix and would be translocated. The presence of the two resulting topoforms could be explained by the subsequent, stochastically determined, behaviour of TM3: if not recognized by the translocon as stop-transfer, it would follow TM2 into the lumen, with generation of the TMs-translocated form (Fig. 3C); if recognized as a stop-transfer sequence, it would integrate into the bilayer, leaving the C-terminus exposed to the cytosol (structure (ii), Fig. 4A). It is also possible that WRB exerts, in addition, a direct effect on TM2, assisting its exit from the lateral gate of the translocon or holding it at the interface between the translocon lateral gate and the surrounding lipids until arrival of TM3.

It must be noted that, in the case of the C-cytosol form, our data do not discriminate between the helix 1 and 3 inserted form, illustrated in Fig. 4A, and alternative 2-TM topoforms (e.g., helix 1 and 2 inserted), which would equally result in exposure of the C-terminal opsin epitope to the cytosol. We favour, however, the TM1 plus 3 model (structure (ii), Fig. 4A): indeed, of the three predicted TMs, TM2 is the poorest one and most likely to be translocated, as is in fact observed in the TMs-translocated form.

Regardless of the topology of the C-cytosol form, the instability of the two aberrant CAML topoforms is most probably due at least in part to the exposure of its TMs (TM2 and/or TM3) to the aqueous environment of the ER lumen or of the cytosol. Within the lumen, the TMs could be engaged by the Hsp70 chaperone, BiP. BiP binds exposed hydrophobic sequences typical of unfolded proteins^23,24^, and previous work has demonstrated that it recognizes translocated TMs for subsequent delivery to the ERAD pathway^25,26^. In the case of exposure of a potential TM to the cytosol, the hydrophobic sequence could be recognized by cytosolic quality control systems, such as the BAG6 scaffold^27,28^. Recognition of unassembled TMs within the bilayer by membrane-embedded proteins could also be involved in driving the aberrant topoforms to degradation^29^.

A number of instances are known, in which poorly hydrophobic TMs of polytopic proteins are transiently translocated into the ER lumen and subsequently integrated into the bilayer once necessary downstream TMs become available^30,31^. In the case of CAML, however, the TMs translocated version does not appear to be a normal biogenetic intermediate, as indicated by the near absence of the 2-G form in cells co-expressing WRB.

Our data further suggest that the two incorrectly inserted CAML forms are generated during the insertion process, and not after correct insertion followed by topological rearrangement: this is indicated by the large proportion of the 0-G form that was generated in the absence of co-expressed WRB; in the 0-G form, the opsin epitope presumably never visited the ER lumen. Thus, the role of WRB is not limited to stabilizing its correctly inserted partner; rather, WRB plays an active role in assisting the correct insertion of the problematic CAML. To be noted, while the *raison d*'*être* of poorly hydrophobic TMs is often explained in terms of function, e.g., in transporters, ion channels, or in oligomers with interacting complementary TMs, the selective advantage of CAML’s problematic TMs is not understood at present. One might speculate that, by linking correct topogenesis to WRB availability, the poorly hydrophobic TMs prevent excessive build-up of CAML, acting as controllers of its intracellular levels.

There are a few other known cases, in which a membrane complex subunit depends on its partner for correct membrane integration, and not simply for post-insertional stabilization, e.g., the Na,K ATPase heterodimer^18^ and the αβ T cell receptor^25^. However, WRB’s role in CAML insertion appears to differ from the above examples, as its stabilizing effect is exerted on CAML in stoichiometric excess. Although the BN-PAGE analysis of the current study suggests that CAML and WRB belong to the same supramolecular complexes, several converging lines of evidence indicate that in different types of cells, endogenous CAML is present in excess over endogenous WRB: (i) direct comparison of WRB and CAML levels in cultured cells and rat liver microsomes indicated a 4–7 fold excess of CAML over WRB^7^; (ii) importantly, in Down Syndrome fibroblasts, in which WRB (the product of a gene located on chromosome 21) is upregulated the expected 1.5 times compared to control fibroblasts, CAML levels are unaffected, indicating that the ratio of CAML to WRB can vary^7^; (iii) the levels of transfected CAML were increased by endogenous WRB, indicating that the endogenous protein facilitates the insertion of excess CAML (this study); (iv) Co-Ip experiments suggested that WRB is quantitatively associated with CAML, while a portion of CAML did not co-precipitate with WRB (this study). For these reasons, we hypothesize that WRB acts catalytically, rather than as stoichiometric complex partner, to assist CAML insertion into the ER membrane. In agreement with this idea, we note that in a study involving the introduction of CAML into yeast cells, the presence of Get1 doubled the amount of the expressed heterologous protein, even though Get1 and CAML do not form a functional complex^2^.

A recent homology search has led to the proposal that WRB/Get1 are members of a newly defined Oxa1 superfamily of membrane proteins, which would comprise, in addition to the known members of the bacterial/mitochondrial/chloroplast YidC/Oxa1/Alb3 family, three different proteins of the eukaryotic ER membrane, including Get1^6^. Proteins of the Oxa1 family function as insertases, both in Sec-dependent and independent modes^32–36^, and assist folding^37,38^ and assembly^39^ of membrane proteins. The client TMs usually feature unfavourable free energy for insertion into the bilayer^26,40–43^, (reviews^44,45^), as is the case for CAML’s TM2 and 3. In line with its membership in the Oxa1 family, Get1 is endowed with an insertase activity that post-translationally escorts TAs into the bilayer^46^. This activity could assist the insertion not only of TA proteins delivered *via* TRC40, but also that of C-terminal TMs of polytopic proteins concomitant with translocation of the C-terminus. Whether WRB, beyond its participation to the dedicated pathway of TA protein insertion, has a more general role in membrane biogenesis, is a tantalizing possibility that deserves further exploration.

## Materials and Methods

### Plasmids

pCLX31.1-FLAGhCAML coding for N-terminally FLAG-tagged human CAML (referred to here as FLAG-CAML)^47^ and pRK5rs-WRB-HA coding for C-terminally HA-tagged human WRB^1^ were gifts from Richard Bram (Mayo Clinic College of Medicine, Rochester, MN) and Fabio Vilardi (Universitätsmedizin Göttingen, Germany), respectively. pEGFPN1 was from Clontech (Mountain View, CA).

pcDNA3-FLAG-NCAML and pcDNA3-FLAG-CAML-NGlyc (referred to here as CAML-NGlyc), coding respectively for the FLAG-tagged CAML N-terminal cytosolic domain and for full-length FLAG-CAML tagged at the C-terminus with the first 19 residues of bovine opsin, were generated from fragments amplified on the pCLX31.1-FLAGhCAML template. For both amplifications, we used a forward primer (5′ GGATCCTCGACCATGGACTAC 3′), which contained a 5′ extrasequence with a BamH1 restriction site (underlined), and which spanned the initiation codon and FLAG epitope of the template. For FLAG-NCAML, amplification was carried out with the Expand High Fidelity PCR System of Roche Applied Science (Penzberg, Germany), using as reverse primer a sequence that spans nts 581–616 of the open reading frame, followed by a stop codon (5′ TCATCGAAAAGAGTCAAATTCTTCTG 3′ - reverse stop codon underlined). The amplified fragment was cloned into the PCR-II vector by TOPO ligation (Thermofisher/Invitrogen, Waltham, MA), and from there subcloned into the BamH1/EcoR1 sites of pcDNA3. For CAML-NGlyc, amplification was done with Q5 high fidelity DNA polymerase (New England Biolabs, Ipswich, MA), using as reverse primer (TCAGTCTACTGTTTTGTTGCTGAATGGTACGTAGAAGTTTGGTCCTTCTGTTCCGTTCATTGGTACTTCAGAGCCCCAATAATC) a sequence spanning the last eight codons of CAML plus a 5′ extension with a sequence complementary to one coding for the first 19 residues of bovine opsin (underlined). The amplified fragment was cloned into pCR-Blunt (Thermofisher), then excised with BamH1 and Spe1 and subcloned into the BamH1 and Xba1 sites of pcDNA3. The sequences of the final plasmids were checked for the absence of errors.

### Antibodies

The following primary antibodies were obtained from the indicated sources: monoclonal antibodies (mAbs) against tubulin (clone B-5-1-2) and against the FLAG epitope (clone M2), Sigma-Aldrich (St Louis, MO); anti-PDI mAbs (clone 1D3), Merck-Millipore (Burlington, MA); anti-GFP polyclonals, MBL International (Woburn, MA); affinity-purified antibodies against the coiled-coil domain of WRB were from Synaptic Systems (Göttingen, Germany); polyclonal antibodies against the SDHB subunit of mitochondrial Complex II and against human calreticulin, mouse mAbs against COX IV, ThermoFisher Scientific; anti-TOM40 mouse mAbs (clone D-2), Santa Cruz (Dallas, TX). Anti-bovine opsin mAb R2–15, affinity purified polyclonal anti-Cnx and anti-CAML were kind gifts of Paul Hargrave^48^, Ari Helenius^49^, and Richard Bram^50^ respectively.

The following secondary antibodies were purchased from the indicated sources: anti-rabbit IRDye 800CW and anti-mouse IRDye 680RD, LI-COR Biosciences (Lincoln, NR); horse radish peroxidase-conjugated anti-rabbit and anti-mouse antibodies, Amersham Bioscences (Buckinghamshire, UK); Alexa 488 anti-rabbit and Alexa 568-anti-mouse, ThermoFisher Scientific.

### Cell culture, transfection, silencing, and microinjection

All experiments were carried out with HeLa cells (checked to be mycoplasma-free), grown in DMEM medium supplemented with FBS, L-glutamine and antibiotics under a 5% CO_2_ atmosphere.

Cells at ~70% confluence were transfected using the JetPei transfection reagent (Polyplus-transfection SA, Illkirch, France) according to the manufacturer’s protocol. Plasmids were used at a 1 μg/mL final concentration. In experiments where cells transfected with one or two plasmids were compared, the amount of DNA was held constant in all samples by using a compensating amount of empty vector. Cells were processed for further treatments or analysis 24 h after transfection.

For silencing experiments, cells at ~16% confluence were transfected with RNAi duplexes using the RNAiMAX Lipofectamine reagent (ThermoFisher/Invitrogen) according to the manufacturer’s instructions. RNAi duplexes (siWRB: s14906 - 5′-GGGUGAUAAGUGUCGCUUUtt-3′ and siSCR: Silencer select negative control RNA; Thermofisher/Invitrogen) were used at a 10 nM final concentration. 6 h after transfection the culture medium was replenished with antibiotics and cells were processed for analysis 72 h after transfection. Transfection of silenced cells with protein-coding plasmids was carried out 48 h after silencing; cells were analysed 24 h thereafter.

Microinjection of pCLX31.1-FLAGhCAML, of pRK5rs-WRB-HA (100 ng/μL) and of pEGFPN1 (5 ng/μL) into the nucleus of HeLa cells was carried out as previously described^51^. The microinjected cells were returned to the incubator for 2–2.5 h before being fixed or further treated with 50 μg/ml CHX (Sigma-Aldrich).

In some experiments, silenced and/or protein overexpressing cells were treated with the proteasome inhibitor MG132 (Merck-Millipore/Calbiochem), dissolved in DMSO, at 10 μM final concentration for 6 h. Control cultures were treated with an equal volume of the vector.

### Carbonate extraction and floatation

Cells grown to confluence on 60 cm^2^ plates were scraped with cold PBS, swollen with hypotonic buffer (1 mM Tris-HCl pH 7.5, 1 mM EDTA, 1 mM KCl, 1 mM NaCl + protease inhibitors) for 10 minutes, restored to isotonicity by the addition of an equal volume of 2 × Isotonic buffer (2 mM Tris-HCl pH 7.5, 0.5 M sucrose, 0.2 mM EDTA + protease inhibitors) and homogenized by passing the cell suspension through a 26 G × 1/2″ needle 100 times. After sedimenting the nuclear fraction by low speed centrifugation (850 g, 10 min at 4 °C), membranes were recovered by centrifugation at 150 000 g for 1 hour at 4 °C. The membrane pellet was resuspended in 100 μL of 0.1 M Na_2_CO_3_ and incubated for 30 min on ice. The suspension was then brought to 2 M sucrose, 0.1 M Na_2_CO_3_ in a final volume of 1 mL. The samples, each deriving from one 60 cm^2^ plate, were then layered under a discontinuous sucrose gradient composed of 2.5 mL of 1.6 M sucrose and 1.5 mL of 0.25 M sucrose, both containing 0.1 M Na_2_CO_3_. The gradients were centrifuged overnight (35 000 rpm, 4 °C, Beckman SW55 rotor) and five 1 mL fractions were collected from the top. Fractions were precipitated with TCA, using Na^+^-deoxycholate as carrier, and analyzed by SDS-PAGE/IB.

### BN-PAGE

Membrane fraction was prepared as described in the previous section, resuspended in a buffer containing 500 mM aminocaproic acid, 50 mM Bis-Tris-HCl, pH 7.0, 1 mM EDTA at ~4 μg protein/μL and solubilized by addition of n-Dodecyl beta-D-maltoside at 0.5% final concentration. After incubation for 15 minutes on ice, samples were centrifuged for 15 minutes at 13000 g at 4 °C and ~80 μg of protein of the supernatant were analyzed by BN-PAGE (6–15% gradient gels) followed by blotting as previously described^52^. The blots were probed for CAML, WRB, mitochondrial complex II and IV and TOM40. The latter three (130, 200, and 340 kDa, respectively) were used as size markers. Primary antibodies were detected by horseradish peroxidase-conjugated secondary antibodies followed by ECL.

### Co-Ip

Hela cells, grown to confluence in a 28 cm^2^ culture dish were collected and homogenized as described in the preceding two sections. The homogenates were supplemented with a Deoxy Big Chaps (DBC) containing buffer (final concentrations: 0.6% [w/v] DBC, 50 mM Hepes-K^+^ pH 7.4, 250 mM sorbitol, 70 mM potassium acetate, 5 mM EDTA, 2.5 mM Mg(OAc)_2_, protease inhibitor cocktail), and the nuclei were sedimented by centrifugation at 800 g for 10 minutes at 4 °C. Equal aliquots of postnuclear supernatant were then used for Co-Ip reactions with either anti-CAML, anti-WRB or non-immune rabbit IgG plus protein G agarose beads to collect the immune complexes. After incubation overnight at 4 °C with mild agitation, the beads were collected, washed and analysed by SDS- PAGE/IB with anti-CAML and anti-WRB antibodies. Comparison of the intensity of the bands in the immunoprecipitates with that of the input sample run and imaged in parallel allowed the calculation of the percent immunoprecipitated material for each protein.

### PNGase F treatment

Transfected cells grown on 10 cm^2^ plates were collected directly in 300 μL of 1x Glycoprotein Denaturation Buffer (GlycoDB, New England Biolabs) and heated to 95 °C for 5 minutes. DNA was shredded by passing the lysates through a 26 G × 1/2″ needle 10 times. Aliquots corresponding to 1/30 of the lysates were digested with PNGase F (New England Biolabs) according to the manufacturer’s protocol. Control samples were treated similarly but without the addition of PNGase F.

### Protease protection assay on SP cells

SP cells were generated essentially as described in^53^. Adherent cells were detached by trypsin followed by Soybean Trypsin Inhibitor (Sigma-Aldrich). After sedimentation, cells deriving from one 60 cm^2^ dish were collected into 6 mL of cold KHM buffer (110 mM K^+^OAc, 20 mM Hepes-K^+^ pH 7.4, 2 mM Mg(OAc)_2_)) and recovered by low speed centrifugation; cells were kept on ice for all subsequent operations. The cell pellets were resuspended in 6 mL KHM buffer, treated with 40 μg/mL Digitonin (Merck-Millipore/Calbiochem) for 5 min, then diluted with 14 mL KHM buffer and centrifuged at 1000 g for 3 minutes at 4 °C. The cells were incubated with 14 mL of Hepes buffer (50 mM KOAc, 50 mM HEPES, pH 7.2) for 10 minutes and recovered again by centrifugation. The pellet of SP cells was resuspended with 50 μL KHM buffer and the O.D. of the suspension at 600 nm was determined. 0.08 OD_600_ units were treated with 0.5 mg/mL PK (Sigma-Aldrich) for 30 min in a reaction volume of 30 μL. Parallel samples without PK or with 0.1% TX100 were used as controls. Digestion was blocked by the addition of 8 mM PMSF. Samples were then denatured with GlycoDB and 10 μL aliquots were digested with PNGase F.

### SDS-PAGE/IB

SDS-PAGE and IB were carried out according to standard procedures. PK-treated samples were analysed on 13% Tris-Tricine SDS gels, and sizes of the PFs were estimated on the basis of the migration of Novex Sharp Pre-stained Protein standards (Thermofisher/Life Technologies). Blots were probed with IR dye-conjugated secondary antibodies, scanned with the Odyssey CLx Infrared Imaging System (LI-COR, Bioscience), and the bands were quantified with Image Studio software (LI-COR, Bioscience).

### Immunofluorescence and imaging

Cells were fixed with 4% paraformaldehyde in 0.12 M Na^+^ phosphate buffer, pH 7.4, and processed for immunofluorescence by standard procedures. TX100 (0.3%) was used as permeabilization agent, except when probing for cytosolically exposed epitopes in SP cells. In this case, the cells, grown and transfected on 12 mm^2^ coverslips, were washed twice with SP buffer (0.25 M sucrose, 2.5 mM Mg(OAc)_2_, 25 mM KCl, 1 μM Taxol, 20 mM Hepes-K^+^ pH 7.4), and incubated for 15 minutes at 26 °C with 25 μM Digitonin diluted in the same buffer. Cells were then washed twice with SP buffer, twice with KHM, and fixed. Duplicate samples of fixed SP cells were processed in parallel in the presence or absence of 0.3% TX100.

Confocal images were acquired using the Carl Zeiss LSM 800 confocal microscope in conjunction with the Axio Observer microscope. Images were acquired using x40 (NA 1.3 oil) or x63 (NA 1.4 oil) magnification Plan-Apochromat lenses. Low magnification images (Fig. 3B) were acquired by using the x40 lens with the 0.5 zoom function. Single confocal sections are shown in the illustrations, which were prepared with Adobe Photoshop software.

## Supplementary information

SUPPLEMENTARY MATERIAL FOR THE WRB SUBUNIT OF THE GET3 RECEPTOR IS REQUIRED FOR THE CORRECT INTEGRATION OF ITS PARTNER INTO THE ER

## References

[1] Vilardi F, Lorenz H, Dobberstein B (2011). WRB is the receptor for TRC40/Asna1-mediated insertion of tail-anchored proteins into the ER membrane. J Cell Sci.

[2] Vilardi F, Stephan M, Clancy A, Janshoff A, Schwappach B (2014). WRB and CAML are necessary and sufficient to mediate tail-anchored protein targeting to the ER membrane. PLoS One.

[3] Yamamoto Y, Sakisaka T (2012). Molecular machinery for insertion of tail-anchored membrane proteins into the endoplasmic reticulum membrane in Mammalian cells. Mol Cell.

[4] Chio US, Cho H, Shan SO (2017). Mechanisms of Tail-Anchored Membrane Protein Targeting and Insertion. Annu Rev Cell Dev Biol.

[5] Mateja A, Keenan RJ (2018). A structural perspective on tail-anchored protein biogenesis by the GET pathway. Curr Opin Struct Biol.

[6] Anghel SA, McGilvray PT, Hegde RS, Keenan RJ (2017). Identification of Oxa1 Homologs Operating in the Eukaryotic Endoplasmic Reticulum. Cell Rep.

[7] Colombo SF (2016). Tail-anchored protein insertion in mammals. Function and Reciprocal Interactions of the two subunits of the Trc40 receptor. J. Biol. Chem..

[8] Rivera-Monroy J (2016). Mice lacking WRB reveal differential biogenesis requirements of tail-anchored proteins *in vivo*. Sci Rep.

[9] Juszkiewicz S, Hegde RS (2018). Quality Control of Orphaned Proteins. Mol Cell.

[10] Shing JC, Bram RJ (2017). Yet another hump for CAML: support of cell survival independent of tail-anchored protein insertion. Cell Death Dis.

[11] Shing JC, Lindquist LD, Borgese N, Bram RJ (2017). CAML mediates survival of Myc-induced lymphoma cells independent of tail-anchored protein insertion. Cell Death Discov.

[12] Rivera Monroy, J. Role of WRB protein in cardiac function. *PhD Thesis*, *Georg-August Universitat Gottingen*, 26–28 (2017).

[13] Nilsson IM, von Heijne G (1993). Determination of the distance between the oligosaccaryltransferase active site and the endoplasmic reticulum membrane. J. Biol. Chem..

[14] Pedrazzini E, Villa A, Longhi R, Bulbarelli A, Borgese N (2000). Mechanism of residence of cytochrome b(5), a tail-anchored protein, in the endoplasmic reticulum. J. Cell Biol..

[15] Hessa T (2007). Molecular code for transmembrane-helix recognition by the Sec. 61 translocon. Nature.

[16] Cymer F, von Heijne G, White SH (2015). Mechanisms of integral membrane protein insertion and folding. J Mol Biol.

[17] Skach WR (1994). Biogenesis and transmembrane topology of the CHIP28 water channel at the endoplasmic reticulum. J Cell Biol.

[18] Beguin P, Hasler U, Beggah A, Horisberger JD, Geering K (1998). Membrane integration of Na,K-ATPase alpha-subunits and beta-subunit assembly. J Biol Chem.

[19] Heinrich SU, Rapoport TA (2003). Cooperation of transmembrane segments during the integration of a double-spanning protein into the ER membrane. EMBO J.

[20] Hedin LE (2010). Membrane insertion of marginally hydrophobic transmembrane helices depends on sequence context. J Mol Biol.

[21] Rapoport TA, Li L, Park E (2017). Structural and Mechanistic Insights into Protein Translocation. Annu Rev Cell Dev Biol.

[22] Blobel G (1980). Intracellular protein topogenesis. Proc Natl Acad Sci USA.

[23] Blond-Elguindi S (1993). Affinity panning of a library of peptides displayed on bacteriophages reveals the binding specificity of BiP. Cell.

[24] Flynn GC, Pohl J, Flocco MT, Rothman JE (1991). Peptide-binding specificity of the molecular chaperone BiP. Nature.

[25] Feige MJ, Hendershot LM (2013). Quality control of integral membrane proteins by assembly-dependent membrane integration. Mol Cell.

[26] Coelho JPL (2019). A network of chaperones prevents and detects failures in membrane protein lipid bilayer integration. Nat Commun.

[27] Hessa T (2011). Protein targeting and degradation are coupled for elimination of mislocalized proteins. Nature.

[28] Payapilly A, High S (2014). BAG6 regulates the quality control of a polytopic ERAD substrate. J Cell Sci.

[29] Guna A, Hegde RS (2018). Transmembrane Domain Recognition during Membrane Protein Biogenesis and Quality Control. Curr Biol.

[30] Lu Y (2000). Reorientation of aquaporin-1 topology during maturation in the endoplasmic reticulum. Mol Biol Cell.

[31] Kanki T (2002). The tenth membrane region of band 3 is initially exposed to the luminal side of the endoplasmic reticulum and then integrated into a partially folded band 3 intermediate. Biochemistry.

[32] Serek J (2004). Escherichia coli YidC is a membrane insertase for Sec-independent proteins. Embo J.

[33] van der Laan M (2004). synthase subunit c is a substrate of the novel YidC pathway for membrane protein biogenesis. J Cell Biol.

[34] Yi L, Celebi N, Chen M, Dalbey RE (2004). Sec/SRP requirements and energetics of membrane insertion of subunits a, b, and c of the Escherichia coli F1F0 ATP synthase. J Biol Chem.

[35] Zhu L, Klenner C, Kuhn A, Dalbey RE (2012). Both YidC and SecYEG are required for translocation of the periplasmic loops 1 and 2 of the multispanning membrane protein TatC. J Mol Biol.

[36] van Bloois E, Haan GJ, de Gier JW, Oudega B, Luirink J (2006). Distinct requirements for translocation of the N-tail and C-tail of the Escherichia coli inner membrane protein CyoA. J Biol Chem.

[37] Nagamori S, Smirnova IN, Kaback HR (2004). Role of YidC in folding of polytopic membrane proteins. J Cell Biol.

[38] Serdiuk T (2016). YidC assists the stepwise and stochastic folding of membrane proteins. Nat Chem Biol.

[39] Jia L, Dienhart MK, Stuart RA (2007). Oxa1 directly interacts with Atp9 and mediates its assembly into the mitochondrial F1Fo-ATP synthase complex. Mol Biol Cell.

[40] Ernst S, Schonbauer AK, Bar G, Borsch M, Kuhn A (2011). YidC-driven membrane insertion of single fluorescent Pf3 coat proteins. J Mol Biol.

[41] Price CE, Driessen AJ (2010). Conserved negative charges in the transmembrane segments of subunit K of the NADH:ubiquinone oxidoreductase determine its dependence on YidC for membrane insertion. J Biol Chem.

[42] Guna A, Volkmar N, Christianson JC, Hegde RS (2018). The ER membrane protein complex is a transmembrane domain insertase. Science.

[43] Shurtleff MJ (2018). The ER membrane protein complex interacts cotranslationally to enable biogenesis of multipass membrane proteins. Elife.

[44] Dalbey RE, Kuhn A, Zhu L, Kiefer D (2014). The membrane insertase YidC. Biochim Biophys Acta.

[45] Kuhn A, Kiefer D (2017). Membrane protein insertase YidC in bacteria and archaea. Mol Microbiol.

[46] Wang F, Chan C, Weir NR, Denic V (2014). The Get1/2 transmembrane complex is an endoplasmic-reticulum membrane protein insertase. Nature.

[47] Holloway MP, Bram RJ (1996). A hydrophobic domain of Ca2+-modulating cyclophilin ligand modulates calcium influx signaling in T lymphocytes. J Biol Chem.

[48] Adamus G, Arendt A, Hargrave PA (1991). Genetic control of antibody response to bovin rhodopsin in mice: epitope mapping of rhodopsin structure. J. Neuroimmunol..

[49] Hammond C, Braakman I, Helenius A (1994). Role of N-linked oligosaccharide recognition, glucose trimming, and calnexin in glycoprotein folding and quality control. Proc Natl Acad Sci USA.

[50] Holloway MP, Bram RJ (1998). Co-localization of calcium-modulating cyclophilin ligand with intracellular calcium pools. J Biol Chem.

[51] Ronchi P, Colombo S, Francolini M, Borgese N (2008). Transmembrane domain-dependent partitioning of membrane proteins within the endoplasmic reticulum. J Cell Biol.

[52] Maltecca F (2008). The mitochondrial protease AFG3L2 is essential for axonal development. J Neurosci.

[53] Wilson CM, Bulleid NJ (2003). Investigation of folding and degradation of mutant proteins synthesized in semipermeabilized cells. Methods Mol Biol.

[54] Tsirigos KD, Peters C, Shu N, Kall L, Elofsson A (2015). The TOPCONS web server for consensus prediction of membrane protein topology and signal peptides. Nucleic Acids Res.

