# Supplementary material for: The WRB Subunit of the Get3 Receptor is Required for the Correct Integration of its Partner CAML into the ER
Source: Sci Rep. 2019 Aug 15;9:11887. doi: 10.1038/s41598-019-48363-2 (PMC6695381; doi:10.1038/s41598-019-48363-2)
Supplement: Supplementary file 1 — SUPPLEMENTARY MATERIAL FOR THE WRB SUBUNIT OF THE GET3 RECEPTOR IS REQUIRED FOR THE CORRECT INTEGRATION OF ITS PARTNER INTO THE ER [file 41598_2019_48363_MOESM1_ESM.pdf]

SUPPLEMENTARY MATERIAL FOR

THE WRB SUBUNIT OF THE GET3 RECEPTOR IS REQUIRED FOR THE CORRECT INTEGRATION OF ITS PARTNER INTO THE ER

Hugo J.F. Carvalho, Andrea Del Bondio, Francesca Maltecca, Sara F. Colombo and Nica Borgese

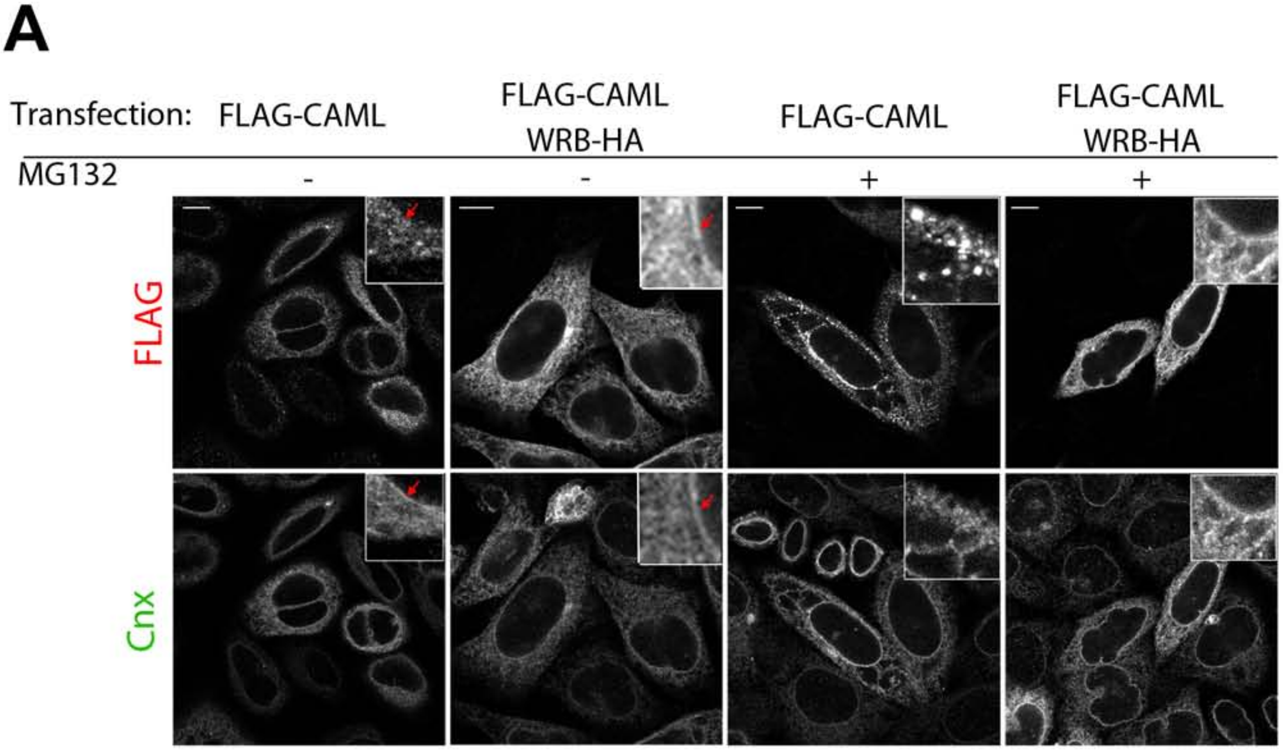

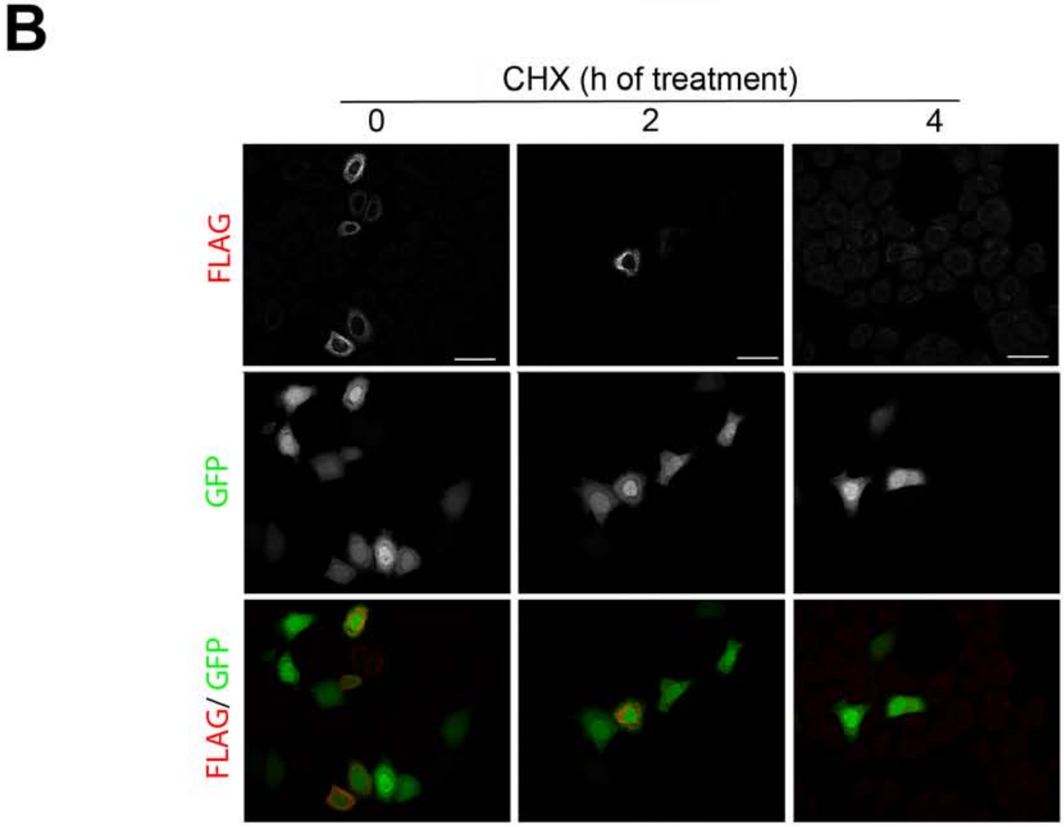

Figure S1 – Confocal images that complement the data of Fig. 2. (A) Single channel images of the merged images of Fig. 2A. **(B)** Representative images used for the time course analysis of Fig. 2D. Scale bars 50μm.

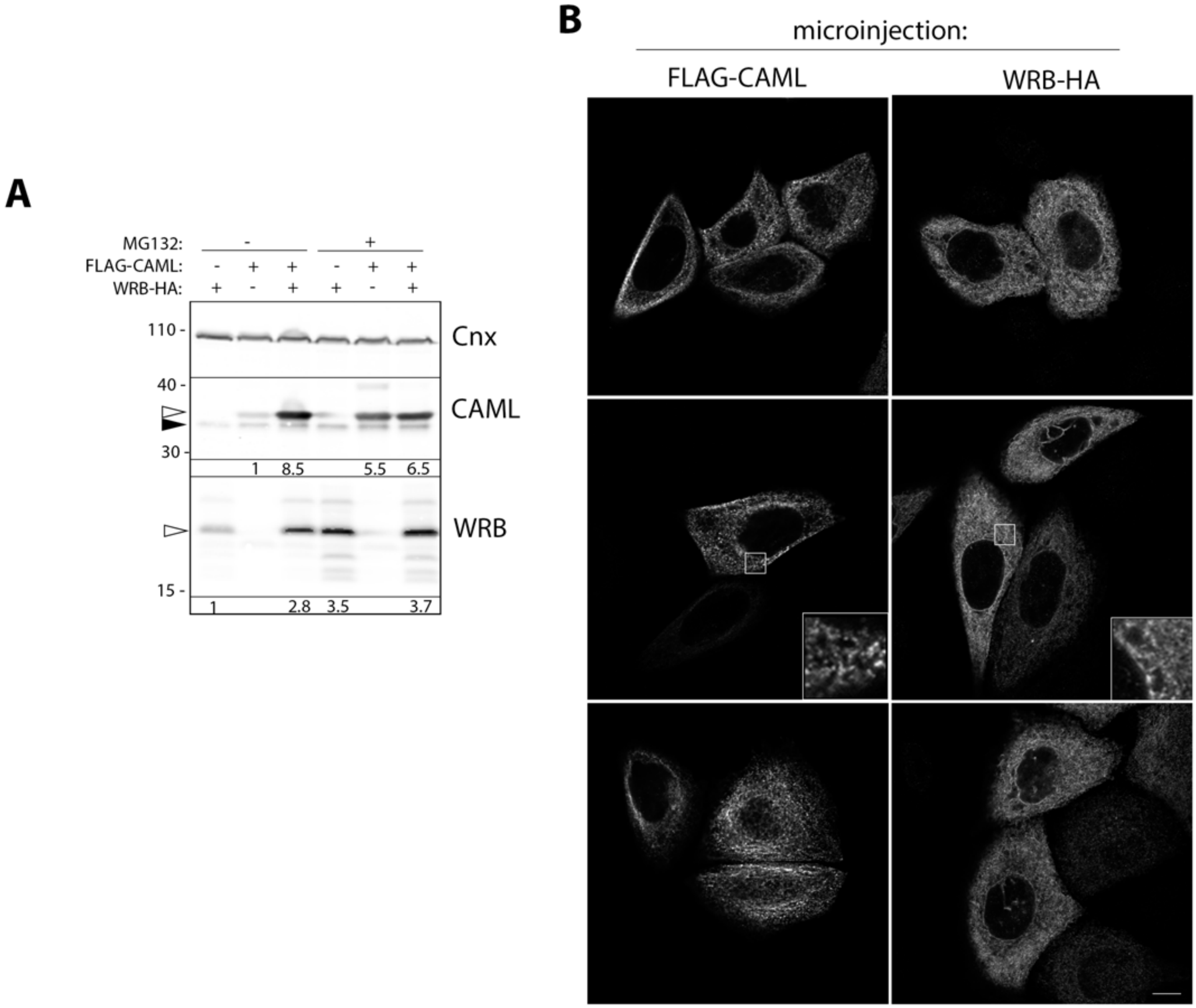

Figure S2 – WRB is stabilized by CAML, but when expressed alone doesn't form ER clusters. (A) The expression of transfected WRB-HA is increased by CAML co-transfection or by proteasome inhibition. HeLa cells transfected with WRB-HA or FLAG-CAML or with plasmids coding for both proteins, as indicated, were treated with MG132 or DMSO for 6h. Lysates were analyzed by SDS-PAGE/IB with the indicated antibodies. The black and white arrowheads indicate endogenous proteins and the tagged transfected forms, respectively. The numbers below the lanes indicate enrichment compared to each construct transfected alone and not treated with MG132. **(B)** WRB-HA, acutely expressed from a microinjected plasmid, is homogeneously distributed in the ER. HeLa cells were microinjected with either the FLAG-CAML or the WRB-HA plasmid, as indicated, and incubated for 2 h before being processed for immunofluorescence with anti-FLAG and anti-HA antibodies. Three different fields for each protein are illustrated. Note that WRB-HA has a typical reticular distribution and is clearly visible on the nuclear envelope, whereas FLAG-CAML forms puncta and is not detected at the nuclear envelope. Scale bars 10μm; insets, x4.

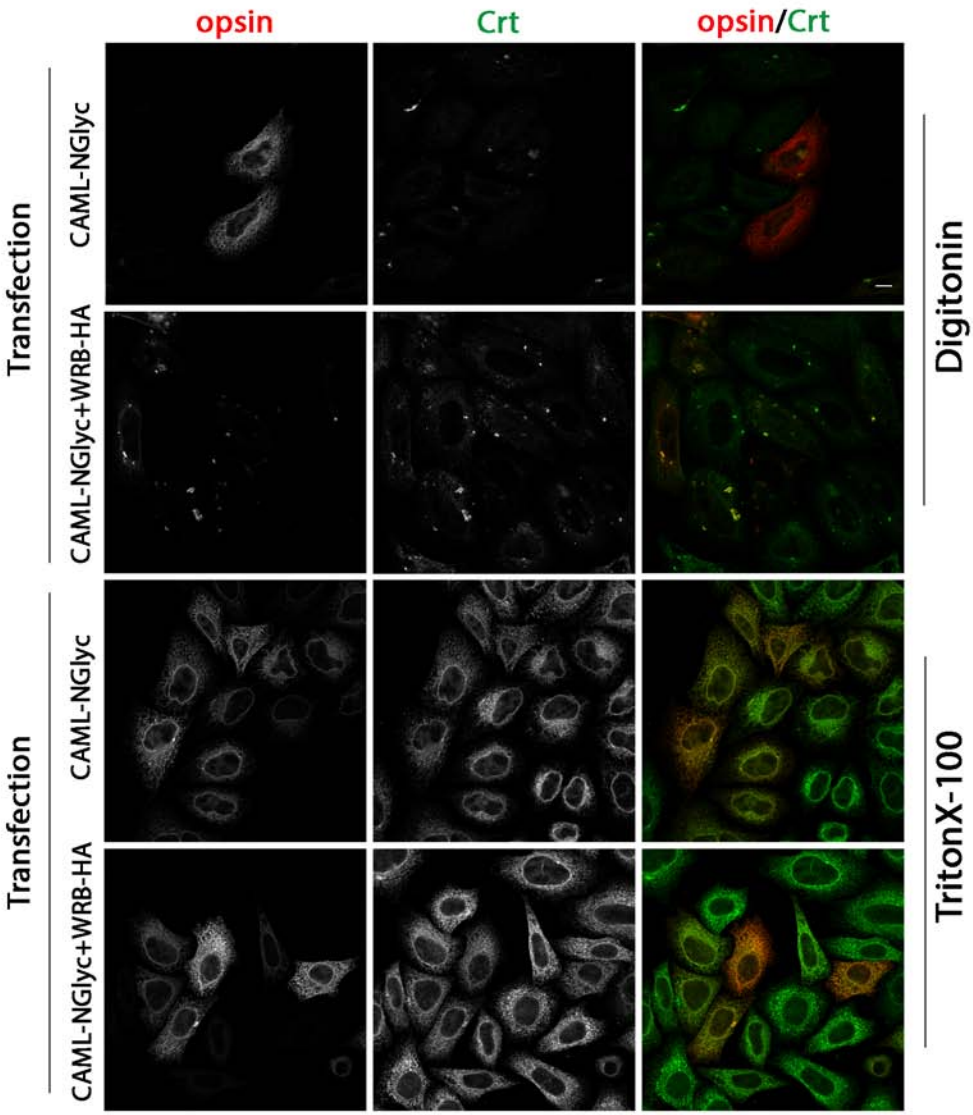

Figure S3- The ER membrane remains intact after digitonin semi-permeabilization. HeLa cells, transfected with CAML-NGlyc either in the presence or absence of WRB-HA, were semi-permeabilized with digitonin before fixation. One set of samples was then processed for immunofluorescence without the addition of other detergents (upper two rows), while the second set was processed in the presence of TX-100 (lower two rows). To visualize the C-terminal opsin epitope of CAML-NGlyc and the endogenous lumenal ER protein calreticulin (Crt), samples were stained with anti-opsin and anti-Crt respectively. Scale bars 10μm.

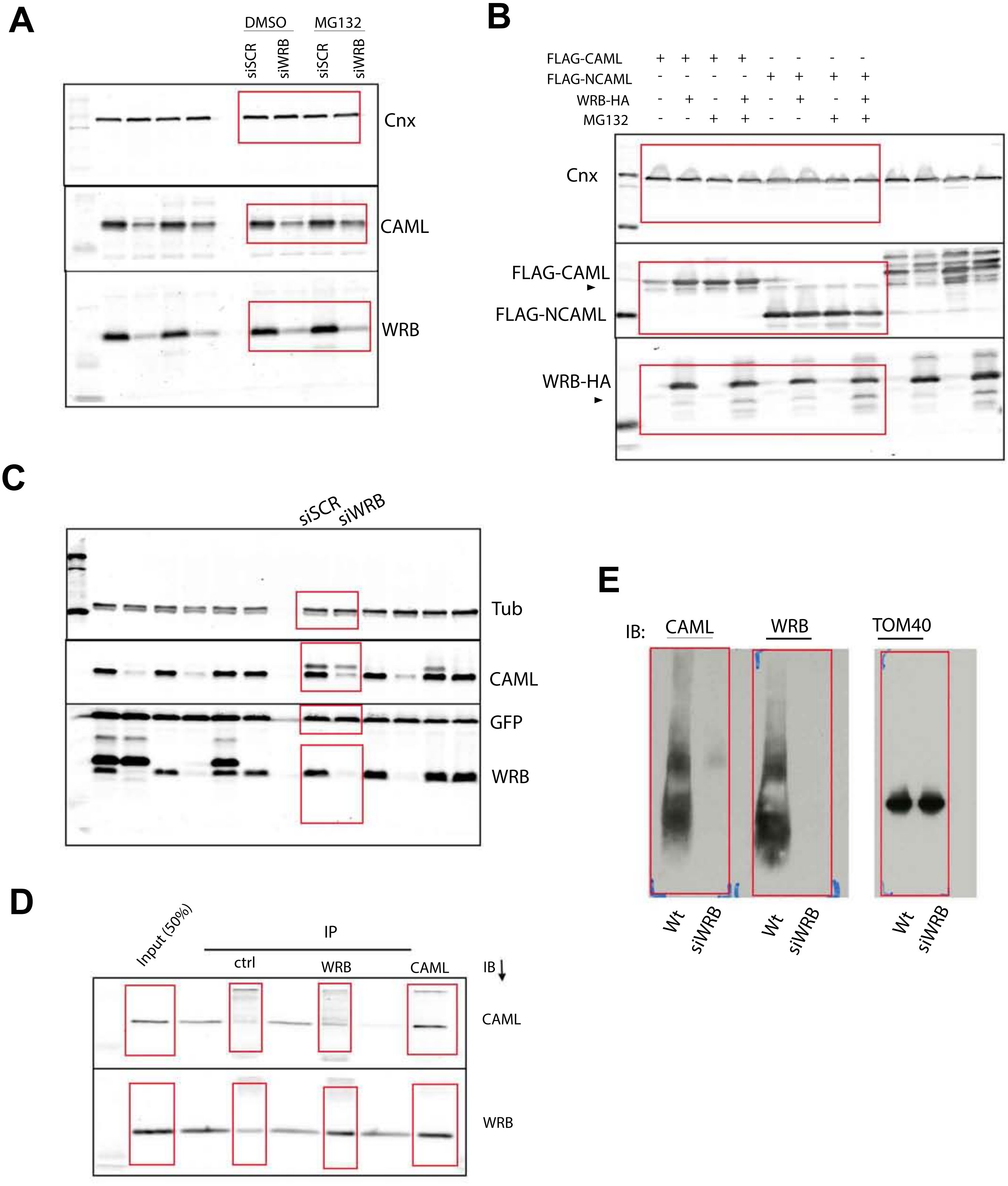

Figure S4 – Original blots related to Figure 1. Regions of the blots shown in the main figure are indicated by red boxes. Lanes present outside of the red boxes are not relevant for this manuscript. Black boxes show the limits of the original membrane. For each panel, individual membrane strips originate from the same blot, which was cut for incubation with different antibodies (indicated on the side). Blots in panels A, B, C and D were scanned with the Odyssey CLx Infrared Imaging System and the Image Studio software was used to adjust the intensity of the fluorescent signal to allow for a better appreciation of the bands of interest. Images were then cropped using Photoshop. Blots in panel E were probed with horseradish peroxidase-conjugated secondary antibodies followed by ECL and exposure to film.

A

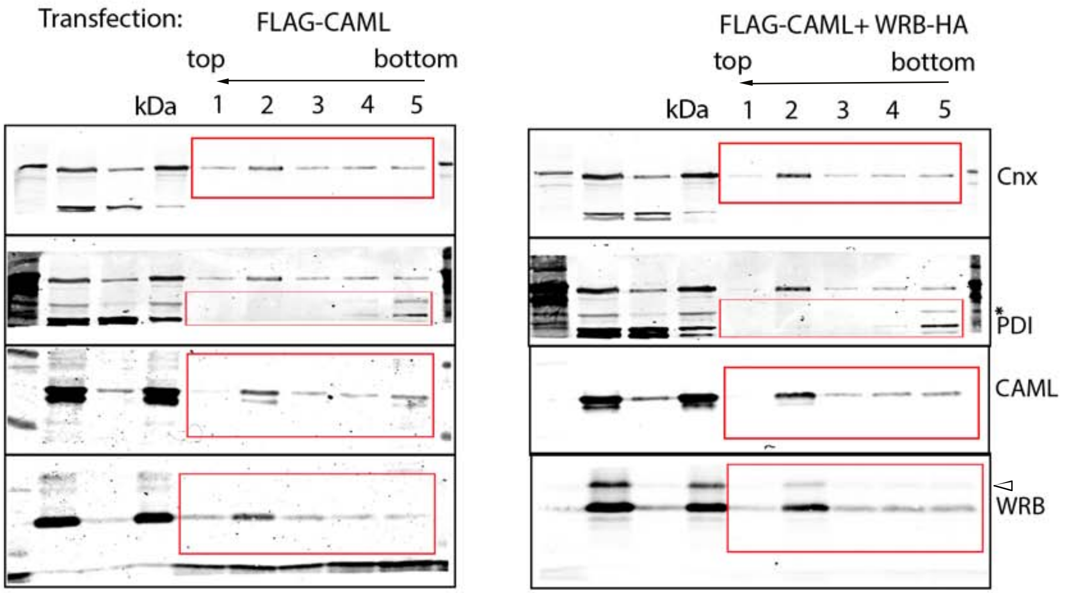

Figure S5 – Original blots related to Figure 2D. Regions of the blots shown in the main figure are indicated by red boxes. Lanes present outside of the red boxes are not relevant for this manuscript. Black boxes show the limits of the original membrane. For each panel, individual membrane strips originate from the same blot, which was cut for incubation with different antibodies (indicated on the side). The upper membranes in both the left and right panels were incubated at the same time with anti-Cnx and anti-PDI antibodies, raised in different species (rabbit and mouse, respectively) and subsequently probed with IR dye-conjugated secondary antibodies with different fluorophores for each species (IR680 and IR800 for mouse and rabbit respectively). Two different intensifications are shown to display the Cnx and PDI bands. Membranes were scanned with the Odyssey CLx Infrared Imaging System and the Image Studio software was used to adjust the intensity of the fluorescent signal to allow for a better appreciation of the bands of interest. Images were then cropped using Photoshop.

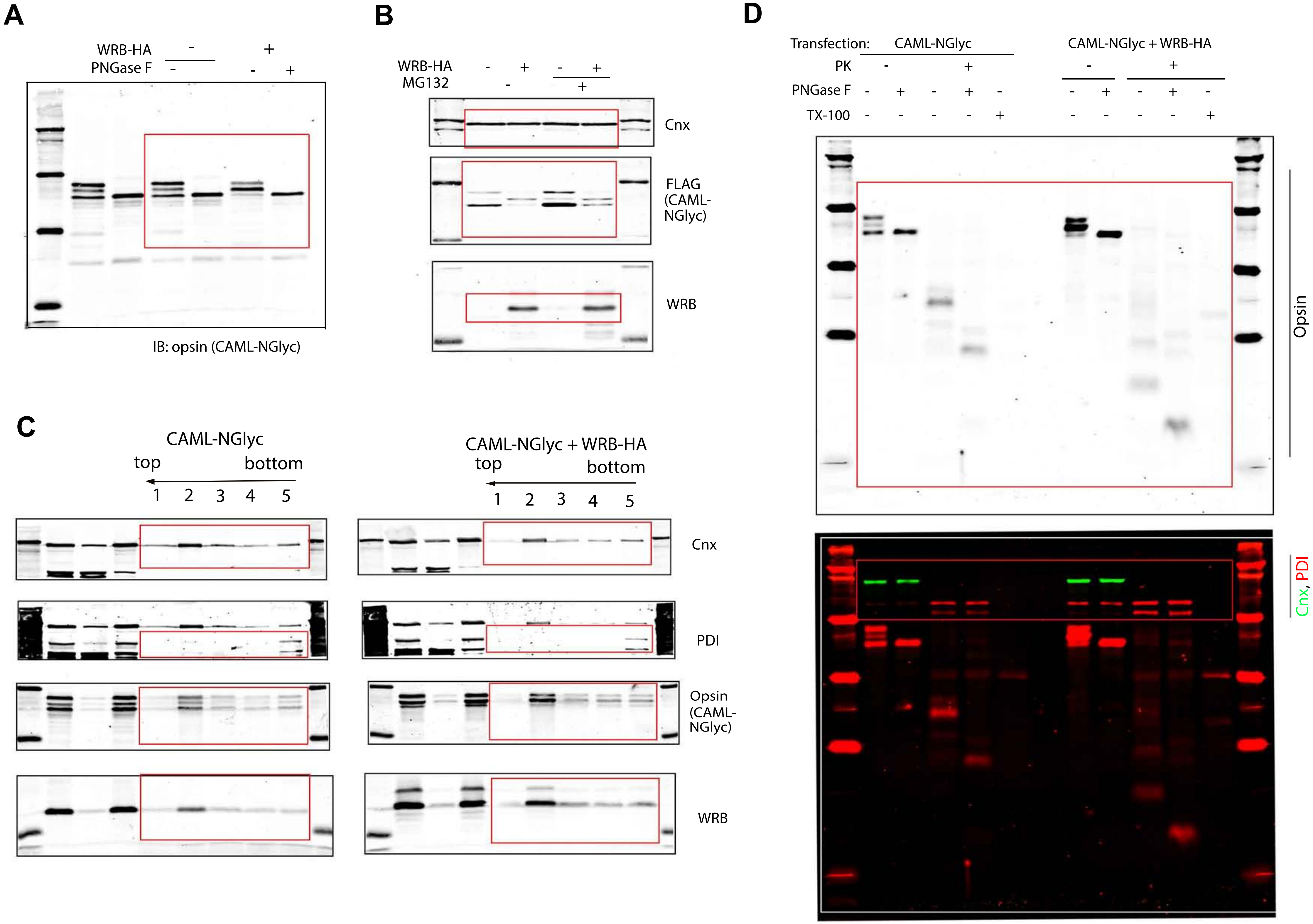

Figure S6 - Original blots related to Figure 3. Regions of the blots shown in the main figure are indicated by red boxes. Lanes present outside of the red boxes are not relevant for this manuscript. Black boxes show the limits of the original membrane. For each panel, individual membrane strips originate from the same blot, which was cut for incubation with different antibodies (indicated on the side). In panel C, the upper two images represent the same membrane strip, which was incubated with anti-Cnx and anti-PDI antibodies, raised in different species (rabbit and mouse, respectively) and subsequently probed with IR dye-conjugated secondary antibodies with different fluorophores for each species (IR680 and IR800 for mouse and rabbit respectively). Two different intensifications are shown to display the Cnx and PDI bands. In panel D, the same membrane was first probed with anti-opsin and after acquisition of the fluorescent signal of a IR680-conjugated secondary antibody (upper blot) the same membrane was incubated simultaneously with anti-Cnx and anti-PDI antibodies raised in different species (rabbit and mouse, respectively) and subsequently probed with IR dye-conjugated secondary antibodies with different fluorophores for each species (IR680 and IR800 for mouse and rabbit respectively - lower strip in colour). Membranes were scanned with the Odyssey CLx Infrared Imaging System and the Image Studio software was used to adjust the intensity of the fluorescent signal to allow for a better appreciation of the bands of interest. Images were then cropped using Photoshop.

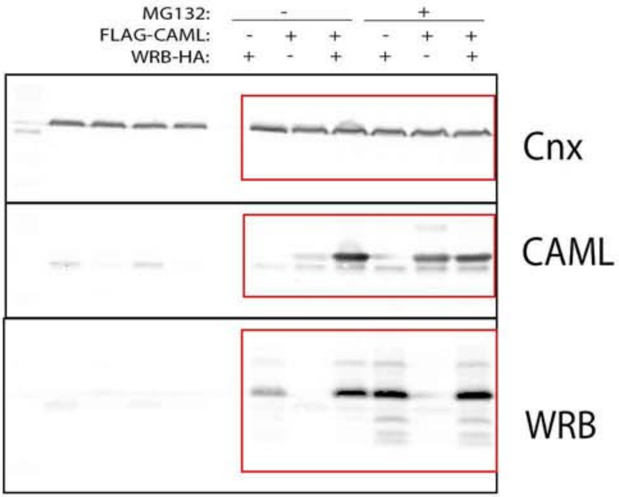

Figure S7 - Original blots related to Figure S2A. Regions of the blots shown in the main figure are indicated by red boxes. Lanes present outside of the red boxes are not relevant for this manuscript. Black boxes show the limits of the original membrane. For each panel, individual membrane strips originate from the same blot, which was cut after blotting for incubation with different antibodies (indicated on the side). Membranes were scanned with the Odyssey CLx Infrared Imaging System and the Image Studio software was used to adjust the intensity of the fluorescent signal to allow for a better appreciation of the bands of interest. Images were then cropped using Photoshop.
